# Phase I trial of afatinib and 3-weekly trastuzumab with optimal anti-diarrheal management in patients with HER2-positive metastatic cancer

**DOI:** 10.1007/s00280-018-3689-2

**Published:** 2018-10-22

**Authors:** Nicolas Martin, Nicolas Isambert, Carlos Gomez-Roca, Rainer-Georg Goeldner, Sylvie Zanetta, Behbood Sadrolhefazi, Hélène de Mont-Serrat, Mario Campone, Jean-Pierre Delord

**Affiliations:** 1 Institut Universitaire du Cancer de Toulouse, Oncopôle, 1 avenue Irène Joliot-Curie, 31059 Toulouse Cedex 9, France; 2 Centre Georges-François Leclerc, 1 rue Professeur Marion BP 77980, 21079 Dijon Cedex, France; 3 Boehringer Ingelheim Pharma GmbH and Co. KG, Global BDS, Birkendorfer Strasse 65, 88397 Biberach an der Riss, Germany; 4 Boehringer Ingelheim (Canada) Ltd./Ltee, 5180 South Service Road, Burlington, ON L7L 5H4 Canada; 5 Boerhinger Ingelheim (France), 12, rue André Huet, 51100 Reims, France; 6 Institut de Cancérologie de L’Ouest site CLCC René Gauducheau, Boulevard Jacques Monod, 44805 Saint-Herblain Cedex, France

**Keywords:** Trastuzumab, Afatinib, HER2-positive cancer, Diarrhea

## Abstract

**Background:**

Trastuzumab is the mainstay of therapy for patients with HER2-positive breast and gastric cancer but resistance frequently occurs. Afatinib, an irreversible oral ErbB family blocker, shows clinical activity in trastuzumab-refractory HER2-positive metastatic breast cancer.

**Materials and methods:**

This phase I study used a modified 3 + 3 dose escalation design to determine the maximum tolerated dose (MTD) of oral once-daily afatinib in combination with 3-weekly intravenous trastuzumab (8 mg/kg week 1; 6 mg/kg 3-weekly thereafter) for patients with confirmed advanced or metastatic HER2-positive cancer.

**Results:**

Of the 13 patients treated, 6 received daily afatinib 20 mg and 7 received 30 mg. One patient who received afatinib 30 mg developed a tumor lysis syndrome and was not evaluable for dose-limiting toxicity (DLT). Two of the six remaining patients receiving afatinib 30 mg and 1 of the 6 patients receiving afatinib 20 mg experienced DLTs (all CTCAE ≥ grade 2 diarrhea despite optimal management) in the first treatment cycle. The most common drug-related adverse events were diarrhea (*n* = 13, 100%), asthenia (*n* = 8, 61.5%), rash (*n* = 7, 53.8%) and paronychia (*n* = 5, 38.5%). No pharmacokinetic interaction was observed. One patient (7.7%) had an objective response (20 mg afatinib cohort). Nine patients (69.2%) experienced clinical benefit.

**Conclusions:**

Despite optimal management of diarrhea including treatment of grade I symptoms, it was not possible to treat the patients above a dose of 20 mg of afatinib daily in combination with 3-weekly trastuzumab. The MTD of afatinib in combination with the recommended 3-weekly dose of trastuzumab was 20 mg daily.

## Introduction

Overexpression of HER2, a tyrosine kinase receptor member of the ErbB family, leads to increased activation of downstream signaling pathways associated with cell proliferation, differentiation, survival and angiogenesis [1]. Trastuzumab, an HER2-targeting monoclonal antibody, is the cornerstone for the treatment of patients with HER2-positive breast and gastric cancer [2]. However, resistance occurs in around 70% of cases and further options are limited [3]. Afatinib is an irreversible oral tyrosine kinase inhibitor (TKI) that targets EGFR, HER2, ErbB3 and ErbB4 transphosphorylation and could potentially overcome resistance to trastuzumab. Results of a phase II trial of afatinib in patients with heavily pre-treated metastatic breast cancer (mBC) resistant to trastuzumab showed promising results [4]. However, afatinib in combination with vinorelbine failed to demonstrate any improvement in progression-free survival (PFS) in comparison to trastuzumab and vinorelbine for trastuzumab pre-treated patients with HER2-positive mBC [5].

Several studies suggest that “vertical” HER2 blockade, using both monoclonal antibodies and TKI, could be a more effective strategy than HER2-directed monotherapies [6]. The association of afatinib plus weekly trastuzumab showed encouraging signs of activity in the single phase I study to date but failed to determine a maximum tolerated dose (MTD) because of a high incidence of severe diarrhea [7]. The optimal schedule for the administration of this association and the prevention of digestive toxicity is still to be determined. Our study was undertaken to determine the MTD of afatinib in combination with 3-weekly trastuzumab and to explore the efficacy, safety and pharmacokinetics of this combination in patients with HER2-overexpressing tumors in a context of optimization of diarrhea management.

## Patients and methods

### Patient population

Patients 18 years old or over with advanced or metastatic cancer that overexpress HER2 (immunohistochemistry 3+ or 2+ with positive gene amplification by FISH) were eligible. For patients with mBC or gastric cancer, prior treatment with trastuzumab in an adjuvant or metastatic setting was permitted, as was prior treatment with lapatinib for mBC in a metastatic setting. Other eligibility criteria were as follows: Eastern Cooperative Oncology Group performance status ≤ 1, life expectancy ≥ 3 months, adequate cardiac and other organ function, and absence of residual toxicity over grade 1 from prior treatment. Patients were not eligible for inclusion if they had received radiotherapy, major surgery, chemotherapy, biological therapy or investigational agents within 4 weeks of first drug administration, had received hormonal treatments within 2 weeks of first drug administration, had relevant cardiovascular abnormalities, known interstitial lung disease, active brain metastases or viral hepatitis, were known HIV carriers, or had any history or presence of poorly controlled gastrointestinal disorders that could affect absorption of the study drug.

The study was carried out in accordance with the principles of the Declaration of Helsinki, the International Conference on Harmonization Harmonized Tripartite Guideline for Good Clinical Practice registration, and local legislation. It was approved by an independent ethics committee. Written informed consent was obtained before each patient’s participation. Trial registration ID: NCT01649271.

### Study design

This was a multicenter, open-label, phase I, dose-escalation study, conducted in three highly qualified phase I cancer centers in France. Patients received continuous oral afatinib once daily in combination with a standard 3-weekly intravenous dose of trastuzumab (loading dose of 8 mg/kg and thereafter 6 mg/kg) as long as they benefited from trial treatment or until they developed undue toxicity. The planned afatinib dose-escalation tiers were 20, 30, 40 and 50 mg per day. The starting dose of afatinib was to be 30 mg per day. Dose escalation followed a modified 3 + 3 design [8], and the MTD was based on dose-limiting toxicities (DLT) observed in the first treatment cycle. DLTs were defined as drug-related adverse events (AE) meeting any of the following criteria according to the National Cancer Institute Common Terminology Criteria for Adverse Events (CTCAE) version 3.0 [9]: uncomplicated grade 4 neutropenia > 7 days, neutropenia associated with fever > 38.5 °C, platelets < 25,000/mm^3^ or grade 3 thrombocytopenia associated with bleeding requiring transfusion, grade ≥ 3 non-hematologic toxicity (except alopecia, incompletely treated nausea, untreated vomiting, or untreated diarrhea), grade ≥ 2 decrease in cardiac left ventricular ejection fraction (LVEF), grade ≥ 2 worsening of renal function as measured by serum creatinine, newly developed proteinuria, or newly developed decrease in glomerular filtration rate, grade ≥ 2 diarrhea persisting for 2 or more days despite supportive treatment, grade ≥ 2 nausea and/or vomiting persisting for 7 or more days despite supportive treatment. An extension cohort was planned at the MTD level of afatinib with trastuzumab following a 3-weekly schedule in a group of 40 evaluable patients. It was also planned to assess the safety of the afatinib MTD dose in combination with the 3-weekly trastuzumab schedule in a 12-patient cohort treated with weekly trastuzumab. However, these two expansion cohorts were never initiated because of the discontinuation of the afatinib development in HER2-overexpressing mBC.

### Management of diarrhea

Diarrhea management is described in Table 1. At the time of afatinib initiation, patients were given anti-diarrheal agents to keep with them at all times and were counseled on how to use a patient diary to record the appropriate use of diarrhea treatment and any improvements or worsening of symptoms. Patients were also advised to drink an adequate amount of fluids to make up for the fluid lost through diarrhea.

**Table 1:** Management of diarrhea

Severity (CTCAE grade)	Description	Intervention concerning afatinib treatment	Specific intervention
Mild (grade 1)	Increase of < 4 stools per day over baseline Mild increase in ostomy output compared with baseline	Continue same dose	200 mg of racecadotril three times a day 4 mg of loperamide to be taken immediately, followed by 2 mg (one tablet) after each loose stool until bowel movements
Moderate (grade 2)	Increase of 4–6 stools per day over baseline Intravenous fluids indicated < 24 h Moderate increase in ostomy output compared with baseline Not interfering with activity of daily life (ADL)	Continue same dose unless grade 2 diarrhea continues for ≥ 2 days, in which case treatment must be interrupted until recovery to grade ≤ 1 followed by dose reduction	Continue loperamide Assess for dehydration and electrolyte imbalance Consider intravenous fluids and electrolyte replacement
Severe (grade 3)	Increase ≥ 7 stools per day over baseline Incontinence Intravenous fluids indicated > 24 h Hospitalization Moderate increase in ostomy output compared with baseline Interfering with ADL	Dose interruption until recovery to grade ≤ 1 followed by dose reduction	See grade 2, plus An infectious process should be ruled out with stool cultures Aggressive intravenous fluid replacement ≥ 24 h Hospitalization to monitor progress Consider prophylactic antibiotics if patient is also neutropenic
Life threatening (grade 4)	Life-threatening consequence (e.g., hemodynamic collapse)	Dose interruption until recovery to grade ≤ 1 followed by dose reduction	See grade 3

### Endpoints

The primary endpoint was MTD of afatinib in combination with 3-weekly trastuzumab and number of patients with DLTs during cycle 1. Secondary endpoints were objective response, best overall response and clinical benefit.

### Safety assessments

Safety was assessed by monitoring AEs, laboratory values, and physical examinations throughout the study. All AEs were graded according to CTCAE version 3.0. Physical examinations, including vital signs, laboratory evaluations, and 12-lead electrocardiogram, were performed at screening and repeated throughout the study. Cardiac LVEF assessment by echography or multigated acquisition scan was performed at screening and every 9 weeks.

### Pharmacokinetic assessments

Pre-dose plasma concentrations (*C*_trough_) were measured at day 8, 15 and 22 for afatinib. Pre-dose plasma concentrations and concentrations at the end of infusion (*C*_eoi_) of trastuzumab were measured at days 1 and 22. Geometrical mean concentrations (gMean) were reported with geometric coefficient of variation (gCV).

### Efficacy assessments

Tumor lesions were assessed using computed tomography within the 28 days preceding the start of treatment and then every 6 weeks thereafter. Response [complete response (CR), partial response (PR), stable disease (SD), or progressive disease (PD)] was evaluated according to Response Evaluation Criteria in Solid Tumors version 1.1 [10].

### Statistical analysis

All analyses were descriptive and exploratory in nature. No formal statistical analysis was planned.

## Results

### Patient population

Between July 2012 and June 2016, 17 patients were enrolled in the study and 13 patients received at least one dose of study medication. Seven patients first received 30 mg (afa30 cohort) and then six patients received 20 mg afatinib per day (afa20 cohort). Median age was 56 years (range 39–65 years). Two patients were male and 11 were female (Table 2). The mean duration of exposure to study medication was 236.6 days (range 21–975 days). Four patients in the afa30 cohort had a dose reduction to 20 mg afatinib. At database lock, all patients had discontinued treatment. Nine patients (69.2%) discontinued it because of disease progression, two patients (15.4%) withdrew due to the occurrence of AEs, one patient (7.7%) refused to continue taking the trial medication, and one patient (7.7%) withdrew from the study for other reasons.

**Table 2:** Baseline demographics and characteristics of patients

	Afatinib 20 mg/day cohort	Afatinib 30 mg/day cohort	Total
Treated set, *n* (%)	6 (100)	7 (100)	13 (100)
Median age, year (range)	61 (56–65)	50 (39–61)	56 (39–65)
Sex, *n* (%)			
Male	2 (33.3)	0 (0)	2 (15.4)
Female	4 (66.7)	7 (100)	11 (84.6)
Tumor type, *n* (%)			
Breast cancer	4 (66.7)	7 (100)	11 (84.6)
Gastric cancer	1 (16.7)	0 (0)	1 (7.7)
Salivary gland cancer	1 (16.7)	0 (0)	1 (7.7)
Median number of metastatic sites, *n* (range)	3 (1–4)	2 (1–5)	3 (1–5)
Metastatic sites, *n* (%)			
Liver	5 (83.3)	1 (14.3)	6 (46.2)
Lung	3 (50)	7 (100)	10 (76.9)
Peritoneum	1 (16.7)	0 (0)	1 (7.7)
Brain	1 (16.7)	0(0)	1 (7.7)
Skin	0 (0)	1 (14.3)	1 (7.7)
Lymph nodes	2 (33.3)	4 (57.1)	6 (46.2)
Bone	2 (33.3)	6 (85.7)	8 (61.5)
Other	1 (16.7)	0 (0)	1 (7.7)
Previous anticancer therapy, (%)	6 (100)	7 (100)	13 (100)
Surgery	6 (100)	7 (100)	13 (100)
Chemotherapy	6 (100)	7 (100)	13 (100)
Radiotherapy	4 (66.7)	5 (71.4)	9 (69.2)
Other	3 (50)	3 (42.9)	6 (46.2)

### DLTs and MTD

One patient in the afa30 cohort developed tumor lysis syndrome during the first course of treatment and was, therefore, not evaluable for the determination of MTD. During the first treatment course, 2 (33.3%) patients in the afa30 cohort experienced DLTs (Table 3). Both were diarrhea (grades 3 and 2) despite optimal anti-diarrheal management. As two out of six evaluable patients experienced DLTs, dose escalation was stopped and more patients were recruited into the afa20 cohort. In that cohort, during the first treatment course, one out of six patients experienced investigator-assessed DLTs. DLTs were diarrhea (grade 3 despite optimal management), elevated blood creatinine (grade 2), and hypokalemia (grade 3) in this single patient. Therefore, the MTD of afatinib in combination with the recommended 3-weekly dose of trastuzumab was determined as 20 mg daily. Across all treatment cycles, three patients (50%) experienced dose-limiting diarrhea in the afa20 cohort and three patients (42.9%) in the afa30 cohort had a dose-limiting decrease in LEVF.

**Table 3:** DLTs in cycle 1

Dose levels		Patients treated, *n*	Patients with DLT in cycle 1, *n*	DLTs
Trastuzumab (mg/kg 3-weekly)	Afatinib (mg/day)			
6^a^	30	7^b^	2	Diarrhea (grades 3 and 2 despite optimal management)
6^a^	20	6	1	Diarrhea (grade 3), serum creatinine increase (grade 2) and hypokalemia (grade 3)

^a^Starting dose 8 mg/kg

^b^Of whom six were evaluable

### Adverse events

All patients had at least one AE (Table 4). The most frequent AEs during treatment were diarrhea (*n* = 13, 100%), asthenia (*n* = 9, 69.2%), rash (*n* = 7, 53.8%), and paronychia (*n* = 6, 46.2%). One patient died during the trial due to worsened general physical status related to disease progression. CTCAE grade 3 events were reported as the worst intensity of an AE for eight patients (61.5%), CTCAE grade 2 for one patient (7.7%), and CTCAE grade 1 for three patients (23.1%). Drug-related AEs were reported for all patients. The most common drug-related AEs were diarrhea (*n* = 13, 100%), asthenia (*n* = 8, 61.5%), rash (*n* = 7, 53.8%) and paronychia (*n* = 5, 38.5%). A total of eight patients (61.5%) had at least one serious adverse event (SAE); diarrhea was the only SAE that occurred in more than one patient (*n* = 2, 15.4%). Three patients, all in the afa30 cohort, had significant decreases in LVEF (below 50%) during the trial. Two patients permanently discontinued the study medication due to AE (one due to cardiotoxicity and one due to diarrhea).

**Table 4:** AEs occurring in ≥ 20% of patients in total, by cohort and overall, system organ classes, and preferred term, sorted by overall frequency in all cycles

System organ class/preferred term	Afatinib 20 mg/day cohort, *n* (%)	Afatinib 30 mg/day cohort, *n* (%)	Total, *n* (%)
Patients	6 (100)	7 (100)	13 (100)
Patients with AEs	6 (100)	7 (100)	13 (100)
Gastrointestinal disorders	6 (100)	7 (100)	13 (100)
Diarrhea	6 (100)	6 (100)	13 (100)
Stomatitis	0	3 (42.9)	3 (23.1)
Nausea	2 (33.3)	1 (14.3)	3 (23.1)
General disorders and administration site conditions	5 (83.3)	7 (100)	12 (92.3)
Asthenia	4 (66.7)	5 (71.4)	9 (69.2)
Infections and infestations	4 (66.7)	6 (85.7)	10 (76.9)
Paronychia	1 (16.7)	5 (71.4)	6 (46.2)
Skin and subcutaneous tissue	4 (66.7)	6 (85.7)	10 (76.9)
Rash	2 (33.3)	5 (71.4)	7 (53.8)
Metabolism and nutrition disorder	3 (50)	4 (57.1)	7 (53.8)
Loss of appetite	1 (16.7)	3 (42.9)	4 (30.8)
Hypokalemia	1 (16.7)	2 (28.6)	3 (23.1)
Investigations	2 (33.3)	4 (57.1)	6 (46.2)
Musculoskeletal and connective tissue disorders	3 (50)	2 (28.6)	5 (38.5)
Muscle spasms	3 (50)	0	3 (23.1)
Respiratory, thoracic and mediastinal disorders	3 (50)	2 (28.6)	5 (38.5)
Hepatobiliary disorders	1 (16.7)	2 (28.6)	3 (23.1)
Nervous system disorders	1 (16.7)	2 (28.6)	3 (23.1)

### Pharmacokinetics

Geometric mean *C*_trough_ values of afatinib at day 8, 15 and 22 were 12.3, 9.92 and 7.08 ng/mL, respectively, (gCV 46%, 65% and 58%) in the 20 mg cohort (Table 5). Mean *C*_trough_ of afatinib at days 15 and 22 was 15.2 and 16.4 ng/mL, respectively (gCV 40% and 43%) in the 30 mg cohort. Mean *C*_trough_ of trastuzumab at day 22 was 30.9 µg/mL (gCV 48%) in the afatinib 20 mg cohort (Table 6) and 32.5 µg/mL (gCV 63%) in the afa30 cohort. Geometric mean *C*_eoi_ of trastuzumab was 212 µg/mL after the first infusion (gCV 14.2%) before starting afatinib and 170 µg/mL and 200 µg/mL (gCV 16% and 28%) after the second infusion in the 20 mg and 30 mg cohorts, respectively. No significant difference was observed for pharmacokinetic parameters of trastuzumab between the two cohorts.

**Table 5:** Pharmacokinetic parameters for afatinib following multiple daily oral administrations in association with trastuzumab 8 mg/kg

	Afatinib 20 mg			Afatinib 30 mg		
	*N*	gMean	gCV (%)	*N*	gMean	gCV (%)
Cycle 1 Day 8 *C*_trough_ ng/mL	5	12.3	46	NA	NA	NA
Cycle 1 Day 15 *C*_trough_ ng/mL	6	9.9	65	4	15.2	40
Cycle 2 Day 1 *C*_trough_ ng/mL	6	7.1	58	4	16.4	43

${C_{trough}}$ predose plasma concentration, *gMean* geometric mean, *gCV%* geometric coefficient of variation

**Table 6:** Pharmacokinetic parameters for trastuzumab after infusion of 8 mg/kg at cycle 1 and 6 mg/kg at cycle 2 in association with daily oral administration of afatinib

	*N*	gMean	gCV (%)
Cycle 1 *C*_eoi_, µg/mL	12	212	14
Cycle 2 Day 1 *C*_trough_, µg/mL			
Afa 20 cohort	7	30.9	48
Afa 30 cohort	4	32.5	63
Cycle 2 Day 1 *C*_eoi_, µg/mL			
Afa 20 cohort	7	170	16
Afa 30 cohort	3	200	28

${C_{trough}}$ minimal concentration, ${C_{eoi}}$ concentration at the end of infusion, *gMean* geometric mean, *gCV%* geometric coefficient of variation

### Efficacy

One patient (7.7%) in the afa20 cohort had an objective response (Table 7). Time to response was 40 days, duration of objective response was 916 days and a clinical benefit was experienced for 955 days. The best response of SD was experienced in 61.5% of patients (*n* = 7 in the afa30 cohort and *n* = 1 in the afa20 cohort). The remaining patients in the afa20 cohort experienced PD (*n* = 3, 23.1%) or the first imaging time point had not been reached before discontinuation (*n* = 1, 7.7%). Nine patients (69.2%) experienced clinical benefit (*n* = 7 in the afa30 cohort and *n* = 2 in the afa20 cohort).

**Table 7:** Best overall response, objective response, and clinical benefit

	Afatinib 20 mg/day cohort, *n* (%)	Afatinib 30 mg/day cohort, *n* (%)	Total, *n* (%)
Patients	6 (100)	7 (100)	13 (100)
Best overall response	6 (100)	7 (100)	13 (100)
Complete response	0 (0)	0 (0)	0 (0)
Partial response	1 (16.7)	0 (0)	1 (7.7)
Stable disease	1 (16.7)	7 (100)	8 (61.5)
Progressive disease	3 (50)	0 (0)	3 (23.1)
Missing^a^	1 (16.7)	0 (0)	1 (7.7)
Objective response			
Yes	1 (16.7)	0 (0)	1 (7.7)
No	4 (66.7)	7 (100)	11 (84.6)
Clinical benefit			
Yes	2 (33.3)	7 (100)	9 (69.2)
No	3 (50)	0 (0)	3 (23.1)

^a^First image time point not reached before discontinuation

## Discussion

The MTD for daily oral afatinib when combined with 3-weekly trastuzumab was 20 mg. As the development of afatinib in HER2-positive metastatic breast cancer was no longer pursued, neither the expansion cohort in patients with mBC nor the cohort intended to evaluate afatinib with the weekly trastuzumab schedule were started.

All DLTs during cycle 1 were diarrhea or its consequences. Diarrhea induced by afatinib is known to be primarily secretory and several mechanisms have been postulated: impaired regulation of chloride secretion [11], mucosal atrophy, altered gut motility, colonic crypt damage, changes in the intestinal microflora and altered colonic transport. Inflammatory or infectious components have also been suggested [12]. In pooled analysis of safety data from phase II and III studies of afatinib, the incidence of all-grade diarrhea was 83.3% and the incidence of ≥ grade 3 diarrhea was 17.9% [13]. A first phase I study which evaluated afatinib in combination with weekly trastuzumab failed to determine a recommended MTD due to excess digestive toxicity. However, the use of antidiarrheal medication was not correctly reported and management may not have been optimal [7]. Management of diarrhea was a major concern in this study. Detailed instructions were given to all patients for the prevention and treatment of diarrhea in accordance with clinical practice for the management of diarrhea in patients treated with afatinib [14]. Although these instructions were carefully observed and reported by all patients, the incidence of diarrhea was 100%. In a recent phase II trial of afatinib in combination with 3-weekly trastuzumab for the treatment of HER2-positive breast cancer in a neo-adjuvant setting, the dosage of afatinib was reduced to 20 mg every second day for the first 2 weeks and a primary prophylaxis with loperamide was obligatory during the first 4 weeks of treatment. Despite these prophylactic measures, the incidence of diarrhea was not significantly reduced (75.4% for grade ≥ 3) [15]. Diarrhea is a common AE of all EGFR TKI. However, its combination with trastuzumab increases incidence of diarrhea. All DLTs in phase I studies of erlotinib or gefitinib in combination with trastuzumab were diarrhea [16, 17]. Moreover, a randomized phase III study of lapatinib or lapatinib in combination with trastuzumab for patients with HER2-positive mBC showed an increased incidence of diarrhea with combination therapy (48% vs 62%) [18].

Median *C*_trough_ of afatinib is known to be higher in patients experiencing high-grade diarrhea [19]. No relevant difference in pharmacokinetic parameters was noted for afatinib and trastuzumab between this cohort and historical ones [20, 21]. Similar findings were observed in the previous phase I study of trastuzumab and afatinib [7]. The incidence of diarrhea with afatinib and trastuzumab seems to be more likely the result of a pharmacodynamic interaction than a pharmacokinetic interaction.

Despite the small sample size, there were indications of clinical antitumor activity with the combined therapy of afatinib plus 3-weekly trastuzumab. One (7.7%) patient had an objective response of 916 days and 9 (69.2%) patients experienced clinical benefit.

Several strategies have been developed in recent years to overcome resistance to trastuzumab. The first approach was dual HER2 blockade with a combination of different HER2-targeted agents. Lapatinib is a dual EGFR/HER2 reversible TKI currently approved for combination with capecitabine or letrozole in patients with HER2-positive mBC. The combination of trastuzumab and lapatinib was associated with improved overall survival (OS) when compared to lapatinib alone [18]. Pertuzumab is a humanized monoclonal antibody which binds to the dimerization domain II of HER2 and blocks ligand-induced HER2/HER3 dimerization. A randomized phase III study conducted in previously untreated patients with HER2-positive mBC revealed improved PFS and OS with pertuzumab in association with trastuzumab and docetaxel or paclitaxel [22]. A recently published pooled analysis of the incidence of diarrhea across all patients treated with pertuzumab and trastuzumab in phase III studies concluded that diarrhea is common but manageable [23]. The combination of pertuzumab plus trastuzumab and taxane-based chemotherapy has received regulatory approval in this setting.

Other TKIs are under development for this subgroup of patients. Neratinib binds irreversibly to the ATP active site of the tyrosine kinase domain of HER2 and blocks signal transduction through EGFR, HER2, and HER4. Neratinib showed promising results in the first open-label study [24], but failed to show non-inferiority when compared to lapatinib plus capecitabine [25]. In another randomized study of paclitaxel plus either neratinib or trastuzumab, patients with central nervous system metastases took longer to develop symptomatic or progressive neurologic disease with neratinib [26]. In these studies, diarrhea was the main side effect with up to 21% of grade 3–4 [27]. A phase III study of neratinib in combination with capecitabine versus lapatinib and capecitabine is underway.

A second approach is the development of an anti-HER2 antibody–drug conjugate. TDM1 consists of DM1, an anti-microtubule agent with highly potent cytotoxicity, bound to trastuzumab. The randomized phase III study EMILIA showed improved PFS and OS with TDM1 in comparison with lapatinib and capecitabine in patients previously treated with a combination of trastuzumab and a taxane, with a better safety profile [28]. TDM1 is currently the treatment of choice in the second-line setting.

The MTD of afatinib in combination with the recommended 3-weekly dose of trastuzumab was 20 mg daily. Diarrhea remains a major concern for the combination, its mechanisms are unclear, and it is probably underexplored in daily clinical practice. Furthermore, new molecules with a better safety and efficacity profile have been approved recently for the treatment of HER2-positive breast cancer, so the development of afatinib for this subgroup of patients has been discontinued.
